# Integrated genetic and transcriptomic analyses suggest a miR-539-3p/SOX9-associated expression pattern in keloids

**DOI:** 10.3389/fcell.2026.1931861

**Published:** 2026-10-21

**Authors:** Zhanpeng Luo, Ling Ge, Lili Chen, Shaoyou Lin, Tianhui Wei, Bo Lu, Zhehu Jin

**Affiliations:** 1 Department of Dermatology, Yanbian University Hospital, Yanbian University Medical College, Yanji, China; 2 Keloid Research Center, Department of Dermatology, Yanbian University Medical College, Yanji, China

**Keywords:** fibrosis, keloid, MiR-539-3p, multi-omics, SOX9

## Abstract

**Background:**

Keloids are fibroproliferative scars characterized by persistent inflammation, aberrant wound healing, and excessive extracellular matrix deposition. Although dysregulated miRNAs have been reported in keloids, their links to disease susceptibility and downstream fibrotic pathways remain incompletely defined.

**Methods:**

We integrated miRNA-QTL-based Mendelian randomization with differential expression analysis of GSE237753 to prioritize keloid-associated miRNAs. Targets of miR-539-3p were screened using miRTarBase, miRDB, and TargetScan, followed by functional annotation and literature-guided prioritization. miR-539-3p and SOX9 expression were assessed by qRT-PCR in eight paired primary normal skin-derived and keloid-derived fibroblast samples, and SOX9 protein expression was examined by immunohistochemistry in 20 independent tissue specimens. SOX9-centered networks were constructed using STRING and GeneMANIA, followed by Gene Ontology and KEGG enrichment analyses. SOX9 mRNA and protein were also measured after miR-539-3p inhibitor treatment.

**Results:**

Mendelian randomization identified nine miRNAs nominally associated with keloid susceptibility, but none remained significant after FDR correction. miR-539-3p showed a nominal inverse association and was the only miRNA overlapping with the differential expression results. In paired primary fibroblasts, both miR-539-3p and SOX9 expression were higher in keloid-derived cells (P = 3.69 × 10^−5^ and P = 1.09 × 10^−5^, respectively). SOX9 immunohistochemistry showed stronger staining in keloid tissue specimens (P = 5.18 × 10^−5^). SOX9 mRNA and protein were higher after inhibitor treatment than after negative-control treatment in three independent experiments per assay. Network analyses linked SOX9 to extracellular matrix organization, connective tissue development, Notch signaling, and TGF-β/Wnt-related remodeling.

**Discussion:**

The genetic analysis focused on genetically predicted circulating miR-539-3p levels, whereas the expression data came from established keloid tissues and derived fibroblasts. These findings reflect different biological contexts, and their different directions are not necessarily contradictory. The increase in SOX9 after miR-539-3p inhibitor treatment warrants further investigation of its potential regulation by miR-539-3p,but does not establish direct targeting in keloid fibroblasts. The expression and network findings provide a rationale for studying whether SOX9 contributes to fibroblast activation and extracellular matrix production in keloids.

## 1 Introduction

Keloids are more than conventional scars. They represent a pathological wound-healing response that remains persistently activated, with excessive fibroblast proliferation, inflammatory signaling, and disproportionate extracellular matrix (ECM) accumulation (Limandjaja et al., 2020; Jeschke et al., 2023). Clinically, lesions expand beyond the original wound boundaries and can cause pain, pruritus and major cosmetic concerns (Limandjaja et al., 2020; Jeschke et al., 2023). Current treatments, including surgery, intralesional corticosteroids, radiotherapy, laser therapy, and cryotherapy, can reduce lesion burden in selected patients, but recurrence remains common (Walsh et al., 2023). These clinical features reflect a persistent pathological remodeling state rather than a simple excess of collagen deposition. Fibroblast activation, inflammatory signaling, vascular changes, epidermal-stromal crosstalk, and extracellular matrix accumulation appear to reinforce one another within the lesion microenvironment (Zhang et al., 2023).

MicroRNAs (miRNAs) are key post‐transcriptional regulators (Bartel, 2004; Guo et al., 2010; Jonas and Izaurralde, 2015) and have been implicated in keloid biology (Liu et al., 2012; Li et al., 2013). However, most reported keloid-associated miRNAs have been identified primarily through differential expression analyses. Although such studies are valuable for discovery, they cannot readily distinguish susceptibility-related signals from secondary molecular changes that arise after lesion formation. Mendelian randomization provides a complementary framework for assessing whether molecular traits are related to disease susceptibility through exposure-associated genetic variation (Sanderson et al., 2022).

Keloid formation is not driven by isolated molecular changes alone, but involves coordinated downstream programs related to fibroblast activation, extracellular matrix remodeling, inflammatory signaling, and abnormal tissue repair (Zhang et al., 2023). However, how upstream miRNA-related signals are connected to these effector programs remains incompletely understood (Liu et al., 2012; Li et al., 2013). An integrative framework combining genetic evidence, disease-state transcriptomic changes, target prediction, and network-based functional annotation may help prioritize biologically relevant miRNA-associated candidates in keloids.

Accordingly, we integrated miRNA-QTL-based Mendelian randomization with public miRNA expression profiling to prioritize candidate miRNAs associated with keloids. Target integration, functional enrichment, network-based annotation, and preliminary experimental validation were then combined to explore plausible downstream targets and their broader biological context. We also assessed SOX9 expression after miR-539-3p inhibitor treatment.

## 2 Materials and methods

### 2.1 GWAS summary statistics

Summary-level genetic association data for plasma miRNA expression were obtained from a previously reported genome-wide association study including 2,178 participants and 2,083 quantified plasma miRNAs (Mustafa et al., 2024). Genetic variants associated with plasma miRNA levels were used as miRNA expression quantitative trait loci (miR-eQTLs) and served as exposure instruments for the subsequent Mendelian randomization analysis. GWAS summary statistics for keloid scar were obtained from the Million Veteran Program (MVP) using PheCode 701.4. The MVP GWAS included 635,969 participants and analyzed 2,068 electronic health record-derived phenotypes (Verma et al., 2024). Because participant availability, phenotype definitions, and case-control classification differed across phenotypes, the effective analyzable population varied among phenotype-specific GWAS analyses. In the present study, the phenotype-specific summary-level association data for PheCode 701.4, including 447,008 analyzable individuals, were used as the outcome dataset for keloid susceptibility.

### 2.2 Mendelian randomization analysis

Two-sample Mendelian randomization (MR) analysis was performed using the Two Sample MR package in R (version 0.6.8) to investigate associations between circulating miRNAs and keloid susceptibility (Hemani et al., 2018). Genetic variants associated with each miRNA were selected as instrumental variables (IVs). To ensure independence among variants, linkage disequilibrium (LD) clumping was conducted using an r^2^ threshold <0.001 within a 10,000 kb window, retaining relatively independent SNPs. Exposure and outcome datasets were subsequently harmonized to align the effect alleles, and palindromic SNPs were removed to avoid strand ambiguity. Instrument strength was assessed using the F-statistics, and all IVs had F values >20. Given that most miRNAs were instrumented by a single SNP, the Wald ratio method was used as the primary MR approach for single-SNP instruments. For miRNAs with multiple available SNPs, the inverse-variance weighted (IVW) method was applied as the main analysis. Nominal P-values were used to identify suggestive MR signals for follow-up, and FDR-adjusted P-values were additionally calculated across the primary MR analyses to account for multiple testing. When multiple SNPs were available, additional sensitivity analyses, including MR-Egger regression and weighted median analysis, were performed where applicable to assess the robustness of the MR estimates.

For miR-539-3p, the MR estimate was based on a single genetic instrument and was obtained with the Wald ratio. MR-Egger regression, weighted median estimation, and leave-one-out analysis with multiple variants could not provide candidate-specific sensitivity evidence for this estimate. Nominal associations, including that for miR-539-3p, were treated as exploratory findings rather than evidence of a causal effect.

### 2.3 Public miRNA-seq dataset and differential expression analysis

Public miRNA-seq count data were obtained from the Gene Expression Omnibus (GEO) dataset GSE237753, a human keloid and normal skin tissue miRNA-seq dataset generated on the GPL20301 Illumina HiSeq 4,000 platform (Mao et al., 2024). According to the GEO record, this miRNA subseries included six human tissue samples, comprising three keloid samples and three normal skin controls, and was therefore suitable for differential miRNA expression analysis. Raw count data (GSE237753_raw_counts.txt.gz) were downloaded and imported into R for downstream analysis. After quality control and filtering of low-abundance miRNAs, differential expression analysis between the keloid and control groups was performed using the DESeq2 package, which models count data using a negative binomial distribution and performs internal normalization based on size factors (Love et al., 2014). For visualization, normalized expression values were transformed to the log2 scale where appropriate. Differentially expressed miRNAs were defined using |log2 fold change| > 1 and FDR-adjusted P < 0.05. Ultimately, the resulting differentially expressed miRNA list was retained for downstream overlap analysis with the nominal MR candidates.

### 2.4 Target gene integration and prioritization

To predict the downstream target genes of the candidate miRNA, target screening was performed by integrating three databases: miRDB, TargetScan, and miRTarBase. First, the target miRNA was searched in each database separately, and the corresponding candidate target mRNA lists were obtained. Among these resources, miRDB predicts functional miRNA targets based on the MirTarget machine-learning algorithm (Chen and Wang, 2020). TargetScan predicts target genes according to the pairing between the miRNA seed region and the 3′UTR of target genes, as well as site conservation and contextual features (Agarwal et al., 2015). miRTarBase was used to supplement predicted targets with curated miRNA-mRNA interactions supported by prior experiments (Huang et al., 2020). After integrating the three resources and harmonizing gene nomenclature, the retained candidate genes were carried forward for functional enrichment and literature-based prioritization. SOX9 was selected for further evaluation based on convergence across the integrated target resources, the curated human hsa-miR-539-3p/SOX9 record in miRTarBase, fibrosis- and keloid-related functional annotation, and its biological relevance to developmental and mesenchymal remodeling programs (Li et al., 2018; Scharf et al., 2019).

### 2.5 Functional enrichment analysis of candidate target genes

The candidate target genes retained after integrating miRDB, TargetScan, and miRTarBase were subjected to Gene Ontology (GO) and Kyoto Encyclopedia of Genes and Genomes (KEGG) enrichment analyses. GO enrichment was evaluated across the biological process (BP), cellular component (CC), and molecular function (MF) categories. KEGG pathway analysis was performed to identify signaling pathways potentially represented by the candidate gene set. Over-representation analysis was conducted using the clusterProfiler package in R (Wu et al., 2021). Terms with FDR-adjusted P-values <0.05 were considered significant. This analysis was used as an initial functional annotation step to support the downstream prioritization of candidate genes.

### 2.6 SOX9-centered interaction network analysis

To further characterize the broader functional context of SOX9, SOX9-centered interaction networks were constructed using STRING (Szklarczyk et al., 2023) and GeneMANIA (Warde-Farley et al., 2010). In STRING, SOX9 was submitted as the query gene, the species was restricted to *Homo sapiens*, the minimum required interaction score was set at high confidence (0.700), and the number of first-shell interactors was limited to 50. In GeneMANIA, SOX9 was used as the query gene to identify genes functionally related to SOX9 based on multiple evidence types, including physical interactions, co-expression, predicted interactions, co-localization, genetic interactions, pathway associations, and shared protein domains (Warde-Farley et al., 2010). Network weighting was set to the automatically selected weighting method, and the maximum number of resultant genes was set to 50. STRING and GeneMANIA were used in parallel because they capture partly overlapping but not identical types of biological relationships, thereby providing complementary views of the SOX9-centered network. STRING-derived and GeneMANIA-derived gene sets were analyzed separately and then subjected to GO and KEGG enrichment analyses using the same workflow described above.

### 2.7 Human specimen collection and primary cell culture

Human keloid tissues and corresponding normal skin tissues were obtained from patients undergoing surgical excision at The Affiliated Hospital of Yanbian University (Yanbian Hospital). The use of human tissue specimens and primary cells was approved by the Medical Ethics Committee of The Affiliated Hospital of Yanbian University (Yanbian Hospital) (approval No. 2024373), and written informed consent was obtained from all participants. Primary normal skin-derived fibroblasts (NFS) and primary keloid-derived fibroblasts (KFS) were used for *in vitro* validation experiments. Cells were maintained in a complete culture medium supplemented with 10% fetal bovine serum and 1% penicillin/streptomycin at 37 °C in a humidified incubator containing 5% CO_2_. Cells were passaged with 0.25% trypsin-EDTA when they reached 70%–80% confluence and were collected during logarithmic growth for subsequent experiments. Samples were assigned to the normal control group and the keloid-derived group according to cell origin.

For qRT-PCR validation, eight matched pairs of primary normal skin-derived fibroblasts (NFS) and keloid-derived fibroblasts (KFS) from eight independent patients were analyzed. Immunohistochemistry included 20 independent tissue specimens: ten normal skin and ten keloid specimens.

### 2.8 RNA extraction and qRT-PCR

Total RNA was extracted from NFS and KFS using TRIzol reagent, according to the manufacturer’s instructions. For miR-539-3p analysis, reverse transcription was carried out with the miRNA first Strand cDNA Synthesis Kit (by tailing A; Vazyme), and quantitative real-time PCR was performed using SuperReal PreMix Plus (SYBR Green; TIANGEN Biotech (Beijing) Co., Ltd.; Cat. No. FP205) according to the manufacturer’s protocol. U6 snRNA was used as internal control. The primer sequences were as follows: 5′-GCG​CGA​TCA​TAC​AAG​GAC​AAT-3′ for hsa-miR-539-3p, 5′- CTC​GCT​TCG​GCA​GCA​CA-3′ for U6 forward, and 5′-AAC​GCT​TCA​CGA​ATT​TGC​GT-3′ for U6 reverse.

For SOX9 mRNA analysis, total RNA was extracted from NFS and KFS using TRIzol reagent and reverse-transcribed using FastKing gDNA Dispelling RT SuperMix (TIANGEN Biotech (Beijing) Co., Ltd.; Cat. No. KR118). Quantitative real-time PCR was then performed using SuperReal PreMix Plus (SYBR Green; TIANGEN Biotech (Beijing) Co., Ltd.; Cat. No. FP205) according to the manufacturer’s protocol. GAPDH was used as the endogenous control. The SOX9 primer sequences were as follows: forward, 5′-GAA​GGA​CCA​CCC​GGA​TTA​CA-3′ and reverse, 5′-GCC​TTG​AAG​ATG​GCG​TTG​G-3'. Relative expression levels were calculated using the 2^−ΔΔCt^ method (Livak and Schmittgen, 2001). Each sample was analyzed in technical triplicate, and the mean ΔCt value from the three technical replicates was used for subsequent 2^−ΔΔCt^ calculation. Statistical comparisons were performed using paired sample-level ΔCt values, and technical triplicates were not treated as independent data points. When appropriate, amplification specificity was confirmed using melt-curve analysis.

### 2.9 H&E staining and immunohistochemistry

Paraffin-embedded tissue sections were used for routine histological assessment and immunohistochemical analysis. For hematoxylin and eosin (H&E) staining, 4-µm-thick sections were first deparaffinized in xylene and rehydrated through a graded ethanol series. The sections were then stained with hematoxylin, differentiated and blued as appropriate, followed by counterstaining with eosin. After dehydration, clearing, and mounting, the sections were examined under a light microscope to assess general tissue architecture, epidermal and dermal morphology, and the arrangement of eosinophilic collagen bundles in the lesional dermis.

SOX9 protein expression in paraffin-embedded tissue sections was assessed by immunohistochemistry. Briefly, 4-µm-thick sections were deparaffinized and rehydrated, followed by heat-induced antigen retrieval using citrate buffer (pH 6.0) or EDTA buffer (pH 9.0), as appropriate. Endogenous peroxidase activity was blocked with 3% hydrogen peroxide at room temperature, and nonspecific binding was inhibited using 5% bovine serum albumin or normal goat serum. The sections were subsequently incubated overnight at 4 °C with the primary antibody against SOX9 (Abways; Cat. No. CY5400; dilution 1:100). After washing with phosphate-buffered saline, the sections were incubated with the appropriate secondary antibody at room temperature, developed with 3,3′-diaminobenzidine, and counterstained with hematoxylin. Following dehydration, clearing, and mounting, the stained sections were examined under an optical microscope and photographed.

Protein expression was evaluated semiquantitatively through an immunoreactive score based on staining intensity and the proportion of positive cells (Remmele and Stegner, 1987). SOX9 immunoreactivity was evaluated primarily in the dermal/lesional area. Epidermal keratinocytes, adnexal structures, tissue folds, edge artifacts, dark deposits, and obvious nonspecific background staining were excluded from scoring. Staining intensity was scored as 0 (negative), 1 (weak), 2 (moderate), or 3 (strong). The percentage of positive cells was evaluated separately and assigned scores of 0 (<5%), 1 (5%–25%), 2 (26%–50%), 3 (51%–75%), or 4 (>75%). The final immunohistochemical score was obtained by multiplying the intensity score by the positive-cell proportion score. All tissue sections were reviewed independently by two pathologists blinded to group assignment.

One image was assessed for each of 20 independent tissue specimens. The two blinded pathologists’ IRS values for each image were averaged to obtain one value per specimen. Individual specimen-level SOX9 IRS values are provided in Supplementary Table S2. Group means and standard deviations were calculated from these specimen values. A two-sided exact permutation test compared the groups while keeping the original and newly added specimens in separate sets.

### 2.10 miR-539-3p inhibitor transfection and SOX9 measurement

Primary fibroblasts were studied under untreated (BL), inhibitor negative-control (NC), and miR-539-3p inhibitor conditions. The hsa-miR-539-3p inhibitor (5′-AAA​GAA​AUU​GUC​CUU​GUA​UGA​U-3′) and inhibitor negative-control oligonucleotide (5′-UCU​ACU​CUU​UCU​AGG​AGG​UUG​UGA-3′) were custom-synthesized and HPLC-purified by General Biol (Chuzhou, China). Both oligonucleotides were transfected at a final concentration of 200 nM using Lipofectamine 2000 (Invitrogen, Thermo Fisher Scientific) according to the manufacturer’s instructions. Briefly, the oligonucleotides and Lipofectamine 2000 were diluted separately in Opti-MEM, combined to form transfection complexes, and added to the primary fibroblasts. Cells were harvested 48 h after transfection for qRT-PCR and 72 h after transfection for Western blotting. Total protein concentration was determined using a bicinchoninic acid (BCA) assay before Western blotting. SOX9 mRNA was measured by qRT-PCR in three independent experiments, with NC set to 1 within each experiment for display as 2^−ΔΔCt^. The NC and inhibitor conditions were compared using a two-sided paired t-test on ΔCt values. SOX9 protein and GAPDH were measured by Western blotting in three independent experiments. SOX9/GAPDH ratios were calculated within each experiment, and NC and inhibitor conditions were compared using a two-sided paired t-test.

## 3 Results

The analytical workflow combined miRNA-QTL GWAS data, keloid GWAS summary statistics, public miRNA-seq profiling, target-resource integration, SOX9-centered network analysis, and experimental validation to select a miRNA-associated candidate for follow-up (Figure 1).

**FIGURE 1:**
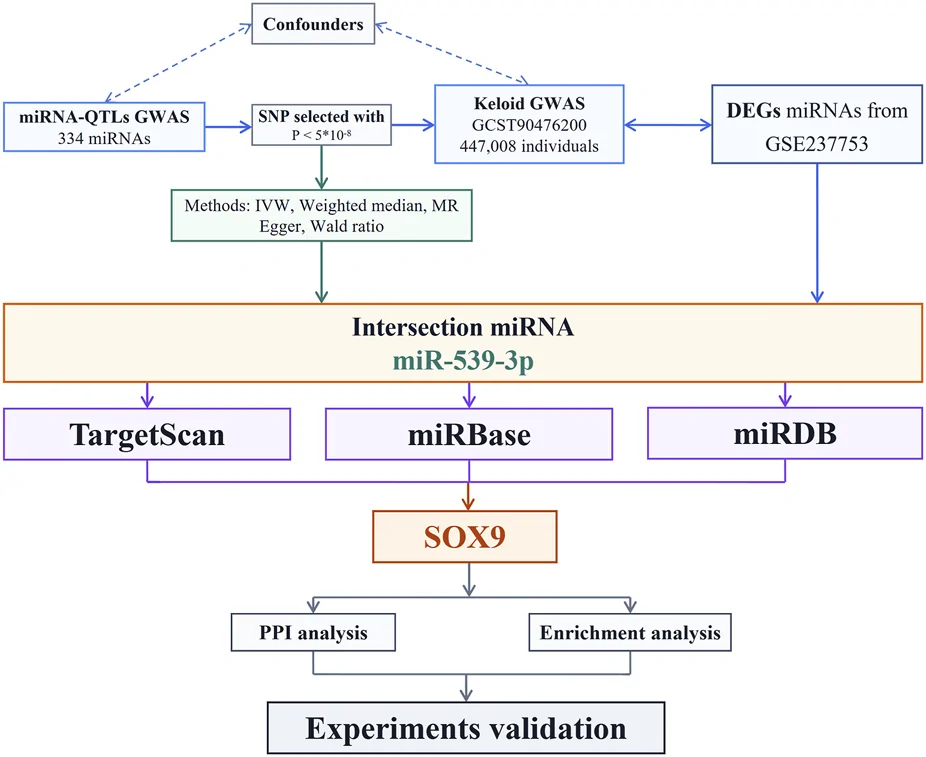
Study design and analytical workflow. miRNA-QTL GWAS data were integrated with keloid GWAS summary statistics for Mendelian randomization analysis. Differentially expressed miRNAs were identified from GSE237753, and the overlapping candidate miRNA, miR-539-3p, was prioritized. Potential downstream targets were screened using target-prediction resources, followed by SOX9- centered PPI analysis, enrichment analysis, and experimental validation.

### 3.1 Integrated MR and transcriptomic screening identified miR-539-3p for exploratory follow-up

The primary MR analysis identified nine circulating miRNAs with nominal associations with keloid susceptibility, including five inverse and four positive associations (Figure 2A). None remained significant after FDR correction. The MR analysis results of all other miRNAs for keloids are provided in Supplementary Table S1.

**FIGURE 2:**
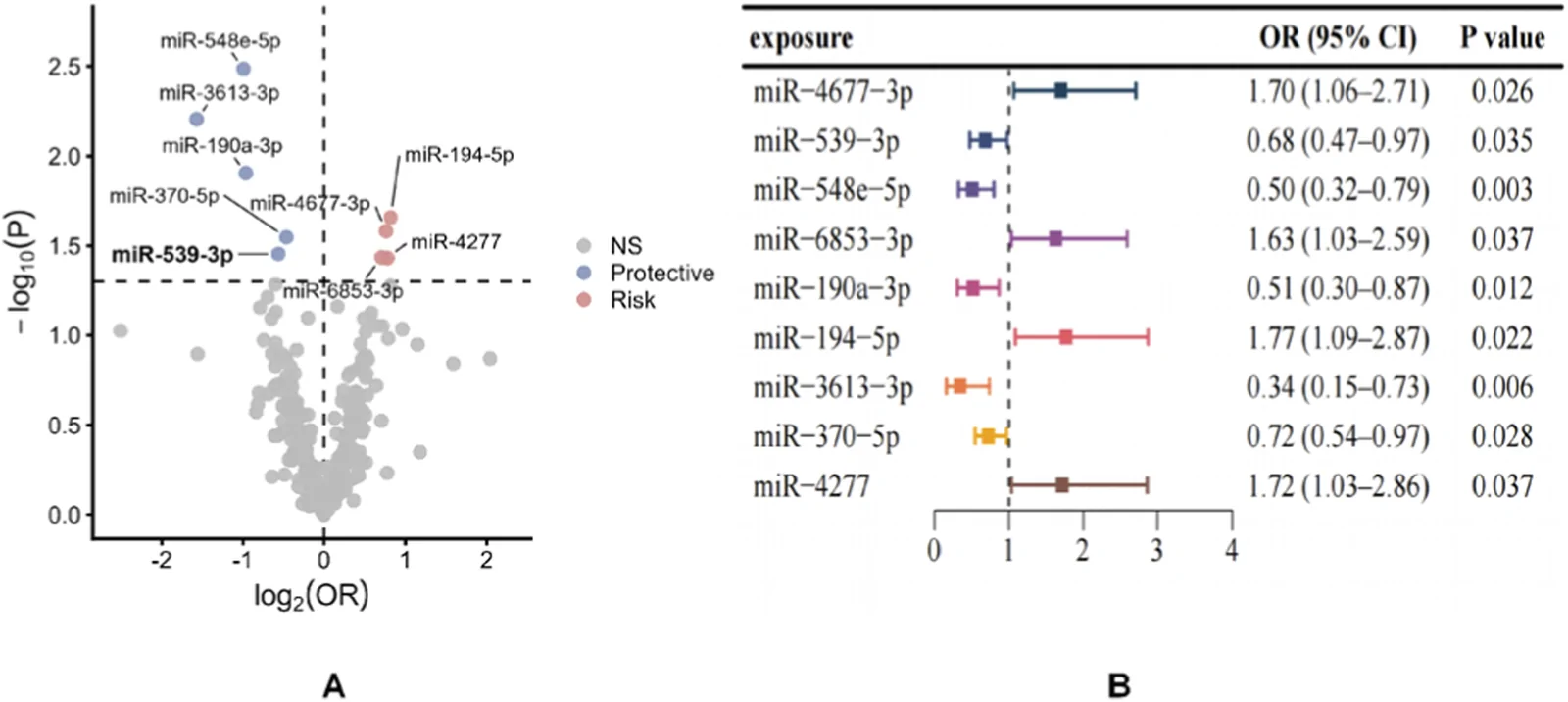
Mendelian randomization analysis of miRNAs associated with keloid susceptibility. **(A)** Volcano plot showing MR associations between genetically predicted miRNA levels and keloid risk. Red and blue dots indicate nominally positive and inverse MR estimates, respectively. **(B)** Forest plot of nine miRNAs showing nominal associations with keloid susceptibility, with odds ratios and 95% confidence intervals. None remained significant after FDR correction.

Among these nominal MR results, miR-4677-3p was associated with higher susceptibility to keloid (OR = 1.70, 95% CI 1.06–2.71, P = 0.026), whereas miR-539-3p showed a nominal inverse association (OR = 0.68, 95% CI 0.47–0.97, P = 0.035; FDR = 0.986). Similarly, miR-548e-5p was nominally associated with lower susceptibility (OR = 0.50, 95% CI 0.32–0.79, P = 0.003), whereas miR-6853-3p was associated with higher susceptibility (OR = 1.63, 95% CI 1.03–2.59, P = 0.037). In addition, miR-190a-3p was inversely associated with keloid susceptibility (OR = 0.51, 95% CI 0.30–0.87, P = 0.012), whereas miR-194-5p was positively associated with susceptibility (OR = 1.77, 95% CI 1.09–2.87, P = 0.022). miR-3613-3p also showed a nominal inverse association (OR = 0.34, 95% CI 0.15–0.73, P = 0.006), and miR-370-5p was associated with lower susceptibility (OR = 0.72, 95% CI 0.54–0.97, P = 0.028). In contrast, miR-4277 exhibited a nominal association with elevated susceptibility (OR = 1.72, 95% CI 1.03–2.86, P = 0.037) (Figure 2B). These estimates are exploratory and do not establish causal protection or risk.

Differential expression analysis of the bulk miRNA-seq dataset identified 44 differentially expressed miRNAs (Figure 3A). Among them, miR-539-3p was significantly upregulated in keloid tissues compared with control tissues (log2FC = 3.29, adjusted P = 4.71 × 10^−6^). These analyses identified miR-539-3p as the only miRNA that met both the suggestive genetic-association criterion and the disease-state differential-expression criterion, thereby providing the rationale for downstream target prioritization (Figure 3B). The overlap was used for candidate selection, not as confirmation of a genetic causal effect.

**FIGURE 3:**
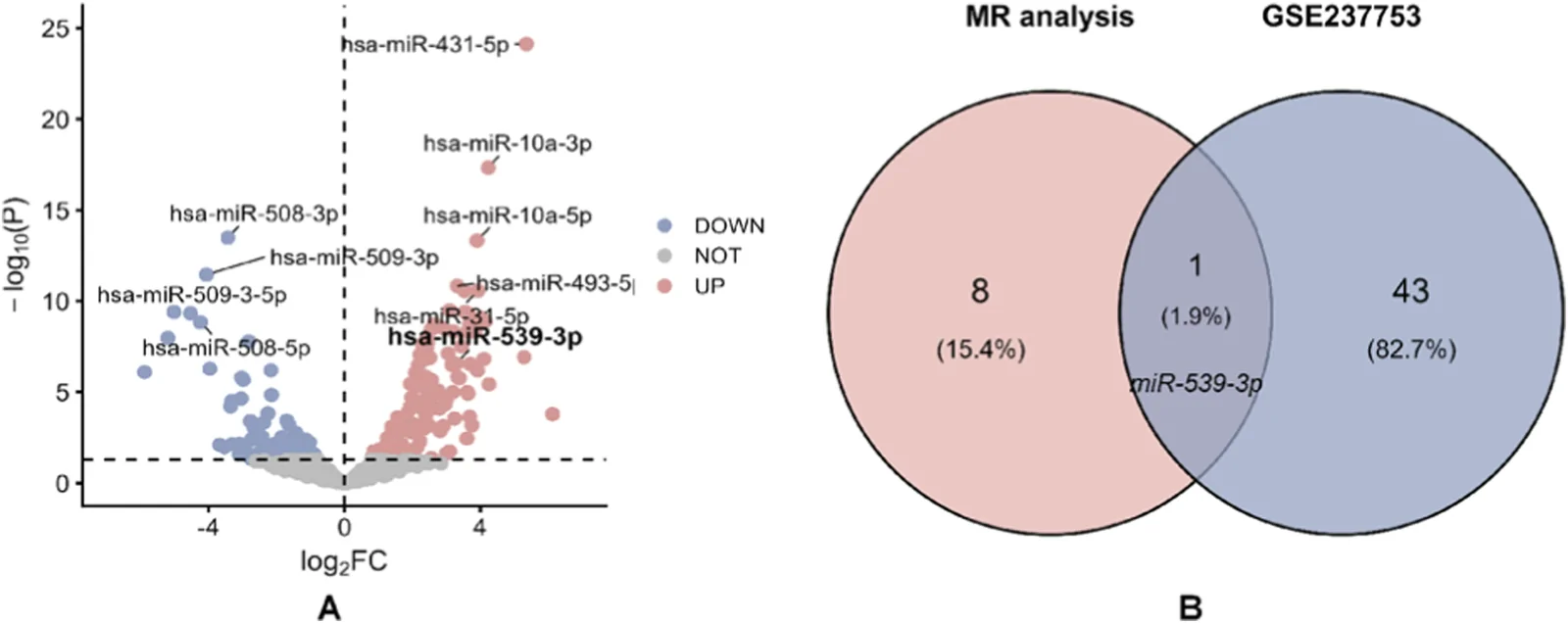
Differential expression analysis and cross-modal prioritization of miR-539-3p. **(A)** Volcano plot of differentially expressed miRNAs in GSE237753, with upregulated and downregulated miRNAs highlighted. miR-539-3p is marked as a candidate miRNA. **(B)** Venn diagram showing the overlap between nominally MR-associated miRNAs and differentially expressed miRNAs, identifying miR-539-3p as the intersecting candidate.

### 3.2 Integrative target screening prioritized SOX9

To identify potential downstream targets of miR-539-3p, we integrated target information from miRTarBase, miRDB, and TargetScan. The Venn analysis identified nine overlapping candidate target genes shared by all three resources (Figure 4A). A miR-539-3p-centered target network was then constructed, including SOX9, CTBP1, NRF1, ARID1A, SRSF2, PCGF3, HS3ST3B1, KPNA2, and MTF2, with SOX9 highlighted as a fibrosis-relevant candidate for further analysis (Figure 4B). Functional enrichment analysis of these overlapping targets showed enrichment in biological processes related to developmental programs, regulation of cell number, stem cell population maintenance, and extracellular matrix organization. Cellular component terms mainly involved nuclear chromatin and nuclear speck, while molecular function terms included transcription corepressor activity, chromatin binding, and DNA-binding transcription factor binding. KEGG pathway analysis further indicated enrichment in transcriptional misregulation in cancer and the PI3K-Akt signaling pathway (Figure 4C).

**FIGURE 4:**
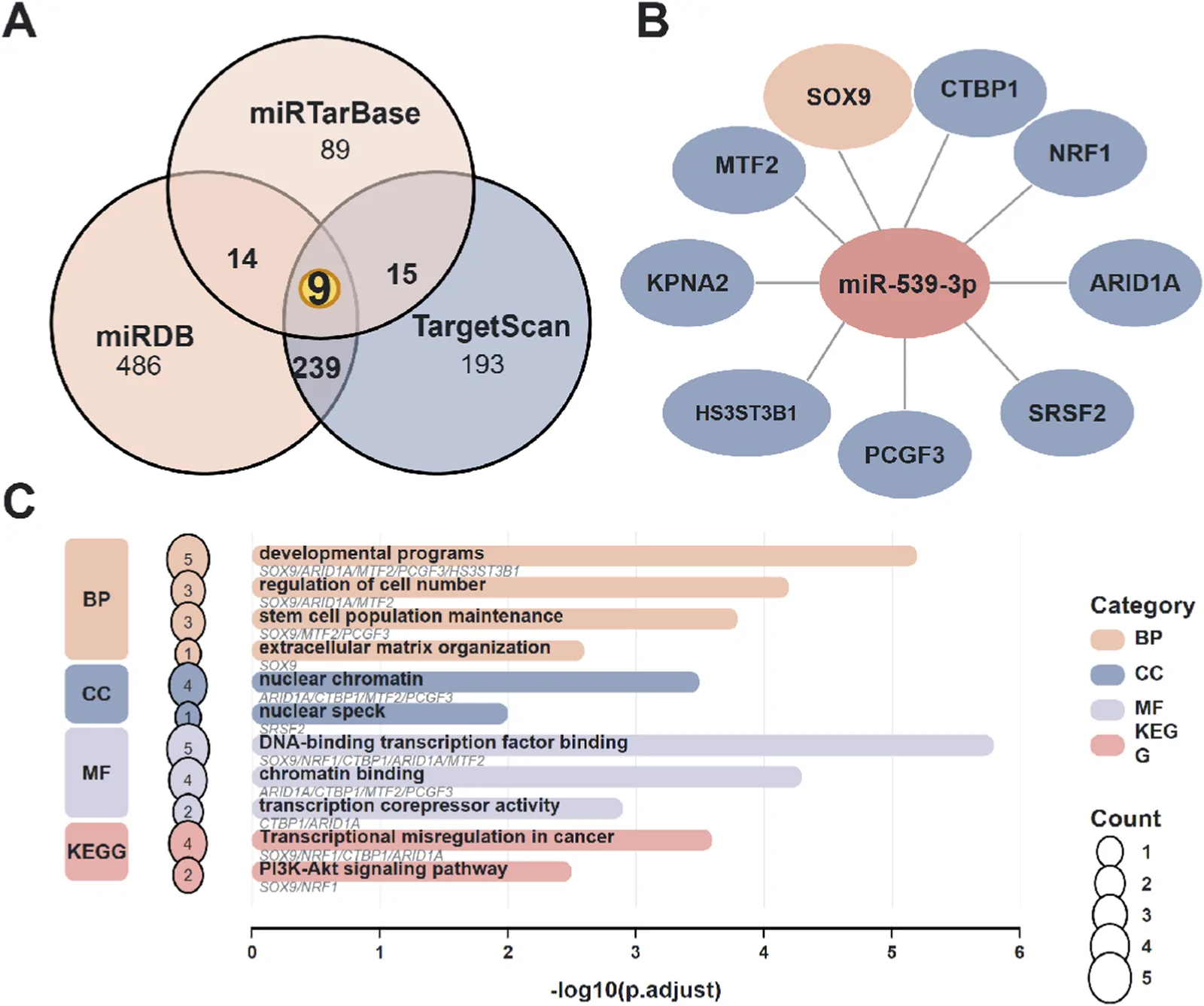
Target integration and functional annotation of miR-539-3p-associated candidate genes. **(A)** Venn diagram showing overlapping predicted or validated targets of miR-539-3p from miRTarBase, miRDB, and TargetScan. **(B)** Network visualization of miR-539-3p and overlapping candidate target genes, highlighting SOX9. **(C)** GO and KEGG enrichment analysis of overlapping target genes.

### 3.3 SOX9-centered interaction network enrichment analysis

For the GeneMANIA-based SOX9-centered network, SOX9 was connected with multiple genes involved in tissue development, extracellular matrix regulation, and signaling-related transcriptional programs (Figure 5A). Functional enrichment analysis of this SOX9-related gene set showed prominent enrichment in biological processes including connective tissue development, extracellular matrix organization, and mesenchyme development (Figure 5B). Cellular component terms were mainly associated with the basal part of the cell and basal plasma membrane, while molecular function terms included DNA-binding transcription factor binding, extracellular matrix structural constituent, and beta-catenin binding. KEGG pathway analysis further indicated enrichment in melanogenesis, Notch signaling pathway, cytoskeleton-related processes, and PI3K-Akt signaling pathway.

**FIGURE 5:**
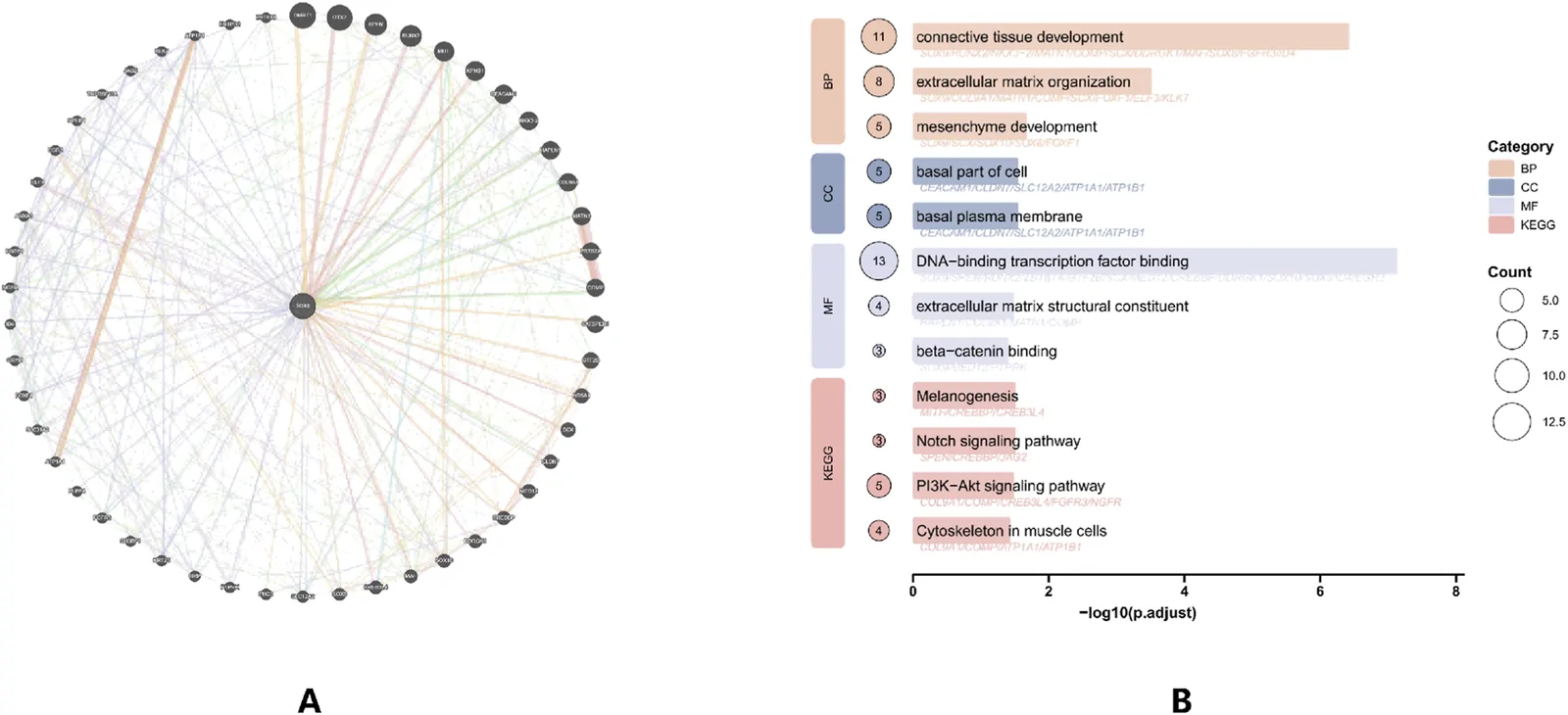
GeneMANIA-based SOX9-centered network and enrichment analysis. **(A)** SOX9-centered interaction network constructed using GeneMANIA. **(B)** GO and KEGG enrichment analysis of SOX9-related genes, showing enrichment in connective tissue development, extracellular matrix organization, mesenchyme development, Notch signaling, and PI3K-Akt signaling pathways.

To further characterize SOX9-associated biological programs, a STRING-based SOX9-centered protein-protein interaction network was constructed (Figure 6A). Enrichment analysis of the STRING-derived SOX9-related genes revealed strong enrichment in connective tissue development, mesenchyme development, and epithelial-to-mesenchymal transition (Figure 6B). The enriched cellular component terms included fibrillar collagen trimer, Wnt signalosome, and collagen-containing extracellular matrix, whereas molecular function terms included co-SMAD binding, SMAD binding, and transforming growth factor beta receptor binding. KEGG pathway analysis highlighted Wnt signaling, melanogenesis, Hippo signaling, Hedgehog signaling, adherens junction, TGF-β signaling, and Notch signaling pathways.

**FIGURE 6:**
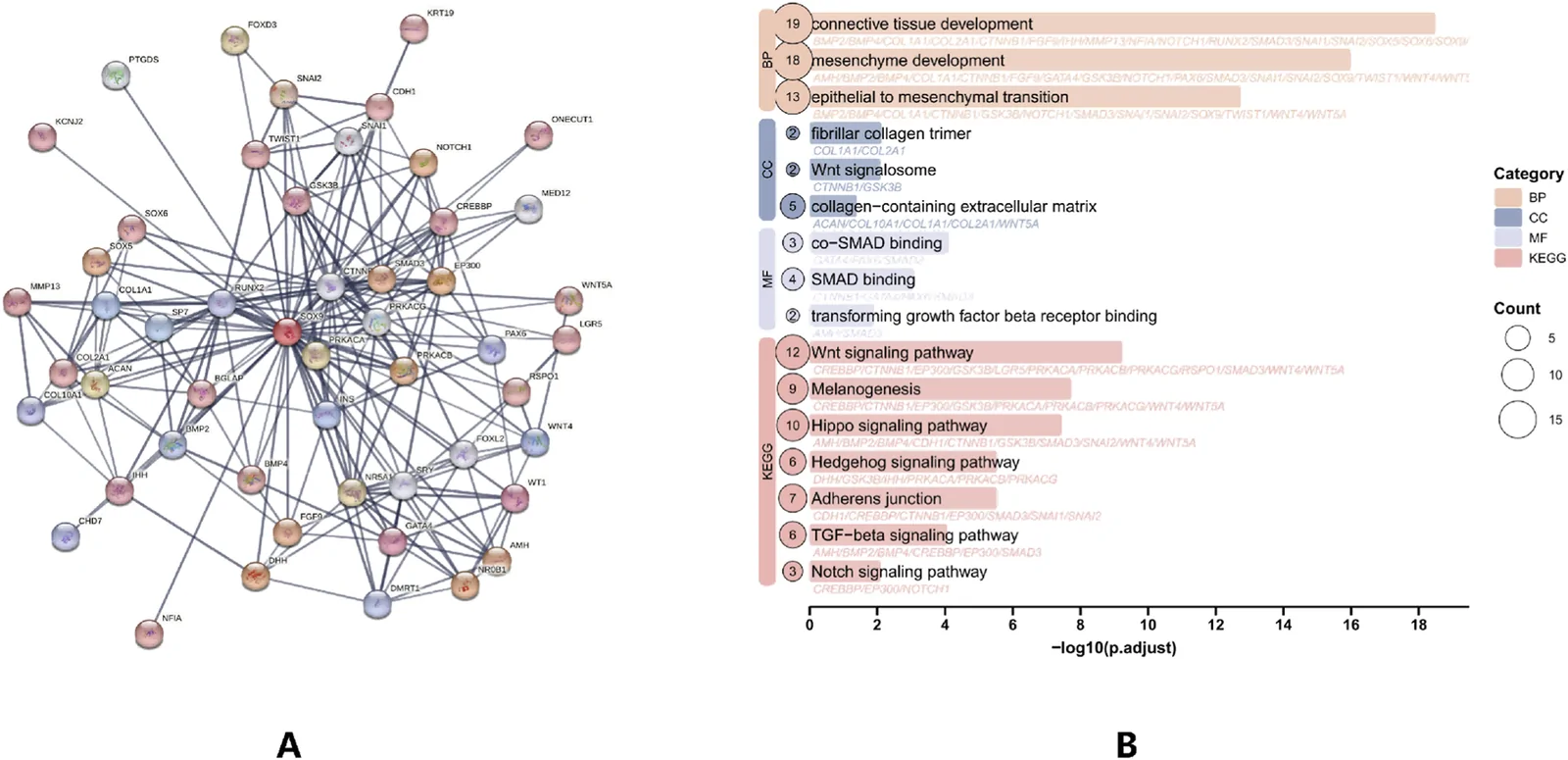
STRING-based SOX9-centered PPI network and enrichment analysis. **(A)** SOX9-centered protein-protein interaction network generated using STRING. **(B)** GO and KEGG enrichment analysis of SOX9-associated genes, highlighting connective tissue development, mesenchymal development, epithelial-to-mesenchymal transition, collagen-containing extracellular matrix, Wnt signaling, TGF-β signaling, and Notch signaling.

### 3.4 miR-539-3p and SOX9 expression in primary cells and tissue

We measured miR-539-3p and SOX9 in eight matched pairs of primary NFS and KFS. Both markers were higher in KFS than in matched NFS (miR-539-3p: P = 3.69 × 10^−5^; SOX9: P = 1.09 × 10^−5^; Figures 7A,B). The arithmetic mean of the eight pair-specific 2^−ΔΔCt^ values was 2.53 for miR-539-3p and 2.61 for SOX9.

**FIGURE 7:**
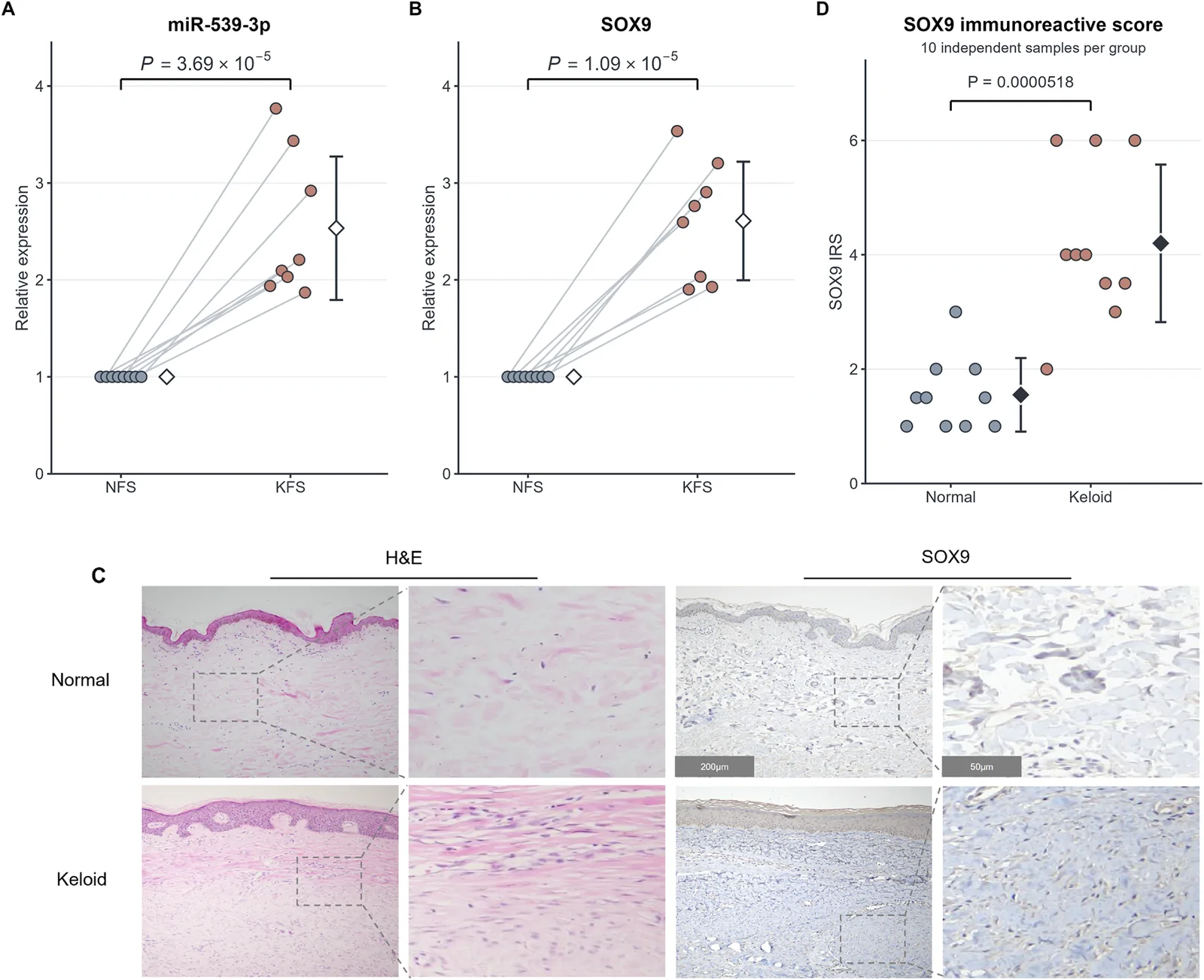
miR-539-3p and SOX9 expression in paired primary fibroblasts and tissue specimens. **(A,B)** qRT-PCR measurements of miR-539-3p and SOX9 in eight matched NFS -KFS primary-cell pairs from eight independent patients. Each normal sample was used as the within-pair reference for the 2^–ΔΔCt^ display. Points and connecting lines represent paired samples; diamonds and error bars indicate mean ± SD. P values compare paired sample-level ΔCt values using two-sided paired t tests. **(C)** Representative H&E and SOX9 immunohistochemistry images of normal skin and keloid specimens. **(D)** SOX9 IRS values from ten normal skin and ten keloid tissue specimens. One image was scored per independent specimen by two blinded readers, and their IRS values were averaged. Circles represent individual specimens; diamonds and error bars show group means ± SD. The P value was calculated using a two-sided exact permutation test that kept the original and added specimen sets separate (see Methods Section 2.9).

H&E staining showed denser collagen deposition and altered dermal architecture in keloid tissue compared with normal skin (Figure 7C). SOX9 immunohistochemistry showed stronger staining in the dermal or lesional compartment of keloid specimens (Figure 7C). Across ten normal skin and ten keloid specimens, mean specimen-level SOX9 IRS values were 1.55 ± 0.64 and 4.20 ± 1.38, respectively (mean ± SD; P = 5.18 × 10^−5^; Figure 7D). The panel shows all 20 specimen-level IRS values.

### 3.5 SOX9 response after miR-539-3p inhibitor treatment

We transfected one group of primary fibroblasts with the miR-539-3p inhibitor and a separate group with the inhibitor negative-control oligonucleotide (NC). We measured SOX9 mRNA by qRT-PCR and SOX9 protein by Western blotting. SOX9 mRNA expression was higher in the inhibitor group than in the NC group across three independent experiments (P = 0.0007; Figure 8A). SOX9/GAPDH protein abundance was also higher in the inhibitor group than in the NC group across three independent experiments (nominal P = 0.0296; Figure 8B).

**FIGURE 8:**
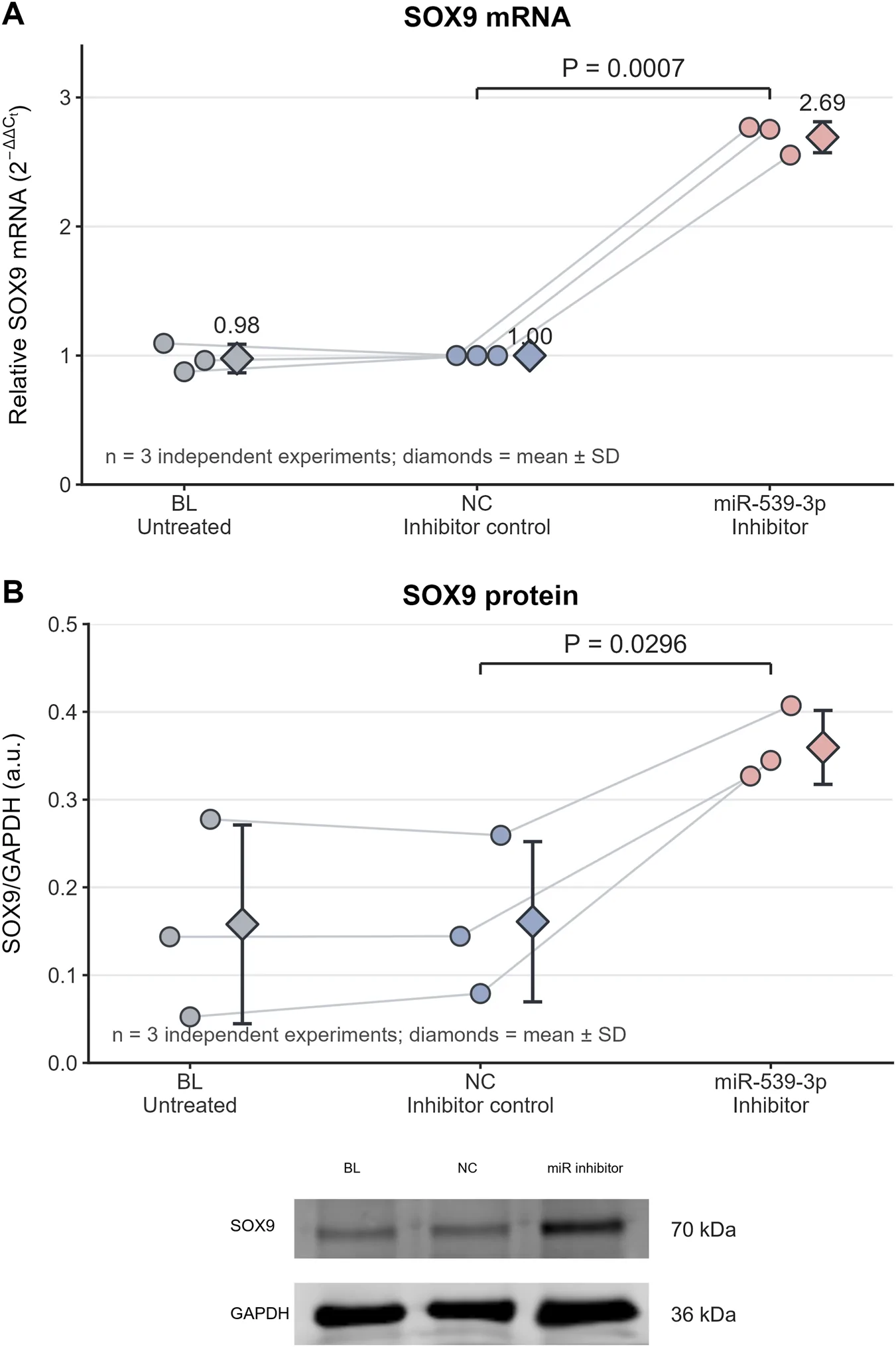
SOX9 expression after miR-539-3p inhibitor treatment. **(A)** SOX9 mRNA expression after untreated (BL), inhibitor negative-control (NC), and miR-539-3p inhibitor treatment. NC was set to 1 within each experiment, and relative expression is shown as 2^−ΔΔCt^. Points and connecting lines show three independent experiments; diamonds and error bars show mean ± SD. The P value compares NC with inhibitor treatment using a two-sided paired t-test on ΔCt values. **(B)** Representative SOX9 and matching GAPDH immunoblots below target/GAPDH densitometry for three independent experiments. Points and lines show paired conditions within each experiment; diamonds and error bars show mean ± SD. The displayed nominal P value compares NC with inhibitor treatment using a two-sided paired t-test on target/GAPDH ratios.

## 4 Discussion

Keloids are chronic fibroproliferative skin lesions in which wound-healing activity persists beyond the normal repair period (Greene et al., 2025). Their clinical behavior—growth beyond the original wound margin, symptoms such as pruritus and pain, and frequent recurrence after conventional treatment—suggests a self-sustaining remodeling state rather than a simple excess of scar tissue (Walsh et al., 2023; Ekstein et al., 2021). Using this framework, we integrated susceptibility-oriented genetic evidence, disease-state miRNA expression, target-resource convergence, SOX9-centered network biology, and preliminary experimental validation rather than relying on expression screening alone.

MicroRNAs are key post-transcriptional regulators that can influence fibroblast activation, inflammatory signaling, extracellular matrix turnover, and tissue remodeling (Bartel, 2009). Although multiple miRNAs have been reported to show altered expression between keloid and normal controls, most prior studies have relied primarily on differential expression, leaving susceptibility-related miRNA signals and disease-state molecular changes insufficiently integrated. Integrating genetic prioritization with disease-state transcriptomic profiling can therefore help distinguish miRNA-associated candidates from broader lesion-associated expression changes. In this study, we combined miRNA-QTL-based Mendelian randomization, public miRNA-seq analysis, target-gene integration, SOX9-centered network annotation, and expanded qRT-PCR/IHC measurements to prioritize keloid-associated candidates. This cross-modal strategy prioritized miR-539-3p as the only miRNA showing overlap between a nominal MR signal and significant disease-state differential expression. Consistent with the public miRNA-seq analysis, qRT-PCR further supported the elevation of miR-539-3p in primary keloid-derived cells. Downstream target-resource integration and literature-guided prioritization identified SOX9 as a fibrosis-relevant downstream candidate. Across eight matched pairs of primary fibroblasts, qRT-PCR showed higher miR-539-3p and SOX9 expression in keloid-derived cells. Immunohistochemistry in 20 independent tissue specimens showed stronger SOX9 staining in keloids, providing a tissue-level protein readout. In three independent experiments per assay, SOX9 mRNA and protein levels were higher after miR-539-3p inhibitor transfection than after negative-control transfection. These findings provide a rationale for SOX9 3′UTR reporter assays and SOX9-specific perturbation or rescue experiments in keloid-derived fibroblasts; they do not establish a direct regulatory pathway.

The different directions of the MR and expression results for miR-539-3p require careful interpretation. Genetically predicted higher plasma miR-539-3p levels were nominally associated with lower susceptibility to keloid, whereas miR-539-3p expression was increased in public keloid samples and in primary keloid-derived cells. This pattern is not necessarily contradictory, because MR estimates reflect inherited variation related to circulating miRNA levels, whereas miRNA-seq and qRT-PCR capture the molecular state of established lesions or lesion-derived cells (Mustafa et al., 2024; Mao et al., 2024). The nominal MR result cannot establish a protective or compensatory effect; none of the miRNA MR associations remained significant after FDR correction. Its value here is as an exploratory candidate-selection signal, alongside disease-state differential expression.

The concurrent upregulation of miR-539-3p and SOX9 in keloid-derived samples should be interpreted in the context of disease-state remodeling rather than as a simple linear miRNA-target relationship. The SOX9 increase after inhibitor treatment adds a treatment-associated observation but does not explain the concurrent baseline elevation. miR-539-3p has been reported to target SOX9 in a chondrogenic differentiation model (Qin et al., 2022); whether this interaction occurs in keloid-derived fibroblasts remains to be tested. The present data therefore do not demonstrate direct regulation of SOX9 by miR-539-3p in keloid cells.

SOX9 was prioritized as a biologically relevant candidate because several lines of evidence link it to lineage remodeling and fibrosis. In keloid tissue, previous gene-expression studies have suggested a partial shift from dermal transcriptional programs toward chondrocytic and osteogenic lineage-associated signatures, accompanied by induction of SOX9 and CBFA1 (Naitoh et al., 2005). This finding places SOX9 within the context of aberrant differentiation programs that may contribute to keloid pathobiology. Evidence from other fibrotic settings further supports this interpretation. For example, TGF-β/Smad signaling has been reported to induce Sox9 in kidney fibroblasts, whereas Sox9 knockdown attenuates experimental renal fibrosis (Li et al., 2018; Raza et al., 2021). Sox9 overexpression has also been associated with fibroblast activation in pulmonary fibrosis, and fibroblast-specific Sox9 deletion after myocardial infarction reduces myocardial scarring and the expression of genes related to extracellular matrix remodeling, proteolysis, and inflammation (Scharf et al., 2019; Gajjala et al., 2021). Together, these studies suggest that SOX9 may participate in fibroblast activation and tissue-remodeling programs across different organs. Although these studies do not demonstrate that SOX9 directly drives keloid fibrosis, they support the biological plausibility of SOX9 as a fibrosis-relevant downstream candidate.

In the SOX9-based STRING and GeneMANIA analyses, SOX9 was connected with genes involved in extracellular matrix organization, connective tissue development, mesenchymal remodeling, and TGF-β/Wnt-related signaling. These processes are closely related to the excessive matrix deposition and fibroblast activation that characterize keloid formation (Sato, 2006; Igota et al., 2013; Deng et al., 2021; Shim et al., 2022). These network results do not demonstrate that SOX9 directly regulates keloid formation, but they place SOX9 within biological processes relevant to fibrotic remodeling (Shim et al., 2022).

A strength of this study is that candidate selection did not rely on differential expression or target prediction alone. Instead, we used a stepwise cross-modal prioritization framework that connected susceptibility-oriented genetic evidence with disease-state miRNA expression, target-resource convergence, SOX9-centered network biology, and preliminary experimental validation. This strategy allowed miR-539-3p to stand out not simply as an upregulated miRNA, but as the only miRNA located at the intersection of suggestive genetic association and keloid-state differential expression. Similarly, SOX9 was prioritized not as an isolated predicted target, but as a fibrosis-relevant transcriptional candidate supported by curated target evidence, keloid-related biological annotation, SOX9-centered network enrichment, and stronger tissue-level protein staining. This design narrowed a broad miRNA discovery space to a focused miR-539-3p/SOX9 candidate relationship for further investigation. The expanded expression measurements and inhibitor response provide additional experimental observations, but they do not establish the direction or directness of regulation.

Several limitations should be considered. The miR-539-3p MR signal was nominal and based on a single SNP, limiting candidate-specific pleiotropy-robust sensitivity analyses (Bowden et al., 2016a; Bowden et al., 2016b). The exposure data came from a European-ancestry miRNA-QTL cohort, whereas the keloid GWAS was multi-ethnic but predominantly European; this composition may limit generalizability (Mustafa et al., 2024; Verma et al., 2024). The public GSE237753 discovery dataset comprised three keloid and three normal skin samples. These primary-cell and tissue-level systems are complementary but not identical. The inhibitor experiment showed increased SOX9 expression. We did not test whether miR-539-3p binds the SOX9 3′UTR or whether altering SOX9 changes fibroblast behavior.

Functional perturbation of miR-539-3p and SOX9 in keloid-derived cells or lesion-relevant models will be necessary to determine whether the observed miR-539-3p/SOX9-associated pattern reflects direct miRNA-target regulation, indirect pathway-level interaction, or a broader remodeling-associated program.

## 5 Conclusion

In conclusion, this study integrated miRNA-QTL-based Mendelian randomization, public keloid miRNA-seq analysis, target-resource convergence, SOX9-centered network annotation, expanded qRT-PCR/IHC measurements, and a miR-539-3p inhibitor experiment to examine a miR-539-3p/SOX9-associated expression pattern in keloids. miR-539-3p emerged as the only miRNA at the intersection of a nominal genetic association and significant disease-state differential expression, and its elevation was further observed in eight paired primary-cell samples. SOX9 was prioritized from the candidate target space and linked by network analysis to extracellular matrix organization, connective tissue development, and TGF-β/Wnt-related remodeling programs. Its expression was higher in paired primary cells and tissue specimens, and SOX9 mRNA and protein increased after miR-539-3p inhibitor treatment. Together, these findings narrow a broad miRNA discovery space to a testable candidate relationship. The genetic evidence remains exploratory, and the proposed regulatory relationship requires direct mechanistic validation.

## Data Availability

The original contributions presented in the study are included in the article/Supplementary Material, further inquiries can be directed to the corresponding authors.
